# A Comparative Study of Radiological, Functional, and Clinical Outcomes of Three-Pin Versus Five-Pin Techniques for Distal Radius Fracture Fixation

**DOI:** 10.7759/cureus.99331

**Published:** 2025-12-15

**Authors:** Sathish Kumar Dake, Meeravali Shaik, Vamsi Karanam

**Affiliations:** 1 Orthopaedics, GITAM (Gandhi Institute of Technology and Management) Institute of Medical Sciences and Research, Visakhapatnam, IND

**Keywords:** 3-pin technique, 5-pin technique, dash score, distal radius fracture, five-pin technique, k-wire fixation, percutaneous pinning, radiological outcomes

## Abstract

Introduction

Distal radius fractures are among the most frequently encountered injuries in orthopedic practice, and their optimal management remains a topic of considerable debate. Percutaneous pinning with Kirschner wires (K-wires) is a widely accepted treatment modality, valued for its minimal invasiveness and cost-effectiveness. However, the ideal number and configuration of pins required to balance fracture stability with potential complications is unclear. This study aims to compare the radiological, functional, and clinical outcomes of three-pin versus five-pin fixation techniques for distal radius fractures.

Methodology

This was a single-center, prospective comparative observational study conducted over a six-month period involving 70 patients with distal radius fractures meeting the inclusion criteria. Patients were allocated to two groups: Group A (n=35) received three-pin fixation, and Group B (n=35) received five-pin fixation. The primary objective was to compare radiological outcomes (radial height, radial inclination, volar tilt), functional outcomes using the Disabilities of the Arm, Shoulder and Hand (DASH) score, and the incidence of complications such as pin tract infection and loosening over a three-month follow-up period.

Results

At the three-month follow-up, the five-pin fixation group demonstrated statistically significant superiority in maintaining radiological parameters, with better preservation of volar tilt (mean 8.2° vs. 5.1°, p=0.04) and radial height (mean 10.5 mm vs. 8.9 mm, p=0.03). However, this radiological advantage did not translate into a statistically significant difference in functional outcomes, as measured by the mean DASH score (14.8 in the three-pin group vs. 13.5 in the five-pin group, p=0.35). Critically, the five-pin group experienced a significantly higher rate of pin tract infections (22.8% vs. 8.5%, p=0.048) and patient-reported discomfort.

Conclusion

The five-pin fixation technique provides enhanced biomechanical stability, resulting in superior maintenance of anatomical reduction in distal radius fractures. However, this benefit is offset by a higher risk of pin-related complications without a corresponding improvement in short-term functional outcomes. The choice between a three-pin and a five-pin construct should be tailored to the individual fracture pattern and patient factors, carefully weighing the need for stability against the risk of iatrogenic complications.

## Introduction

Distal radius fractures represent approximately 15%-20% of all fractures treated in emergency departments, occurring commonly in both young active individuals and elderly patients with osteoporosis [1,2]. The primary goal of surgical intervention is to restore anatomical alignment, enabling early wrist mobilization and return to function. Persistent malalignment predisposes one to post-traumatic arthritis, stiffness, and reduced grip strength [3,4]. Percutaneous pinning with Kirschner wires (K-wires) remains a widely accepted treatment modality, valued for its minimal invasiveness and cost-effectiveness [5,6]. However, consensus on the optimal number and configuration of K-wires remains elusive.

The three-pin technique is popular due to its simplicity and lower complication rates, but critics suggest it may inadequately resist torsional forces, particularly in dorsally comminuted or intra-articular fractures [7]. Conversely, the five-pin technique, theoretically, provides enhanced multi-planar stability through additional fixation points [8]. Despite these theoretical advantages, additional pins may increase risks of neurovascular injury, pin tract infection, patient discomfort, and increased operative time [9,10]. This creates a clinical dilemma: whether enhanced stability justifies the increased complication risk. The current literature lacks direct comparative evidence on radiological stability, functional outcomes, and complication profiles between three-pin and five-pin K-wire constructs for distal radius fractures.

This prospective comparative study aimed to compare radiological outcomes (radial height, radial inclination, volar tilt) between three-pin and five-pin fixation techniques, assess functional outcomes using the Disabilities of the Arm, Shoulder and Hand (DASH) score, and document and compare complication rates between both techniques over a three-month follow-up period.

## Materials and methods

Study aim and objectives

The aim of this study was to compare the clinical efficacy and safety profile of three-pin versus five-pin K-wire fixation techniques in adults with closed distal radius fractures. The specific objectives were to compare radiological outcomes (radial height, radial inclination, and volar tilt) between three-pin and five-pin K-wire fixation techniques at the three-month follow-up, to assess and compare functional outcomes using the DASH questionnaire between the two fixation methods at the three-month follow-up, and to document and compare the incidence and types of complications (including pin tract infection, pin loosening, and soft tissue complications) between the two techniques during the study period.

Study design

This was a single-center, prospective comparative observational study conducted within the Department of Orthopedics, GITAM Institute of Medical Sciences & Research (GIMSR), Visakhapatnam, between January and June 2025. The study followed the STROBE (Strengthening the Reporting of Observational Studies in Epidemiology) guidelines for reporting.

Patients were assigned to one of two treatment groups using a sequential, non-randomized allocation method: Group A received three-pin K-wire fixation, and Group B received five-pin K-wire fixation. Patients were allocated alternately based on their order of presentation to the outpatient clinic. While this sequential allocation ensured equal distribution between groups, we acknowledge this represents a quasi-experimental design rather than a true randomized controlled trial, which may introduce allocation bias. This study was not registered as a clinical trial as it employed a non-randomized comparative design evaluating two established surgical techniques already in routine clinical practice.

Research question and null hypotheses

The research question guiding this investigation was whether five-pin fixation provides superior radiological and functional outcomes compared to three-pin fixation in adults with closed distal radius fractures, without significantly increasing complication rates. To address this question, three null hypotheses were formulated and tested: first, that there is no statistically significant difference in radiological parameters (radial height, radial inclination, volar tilt) between three-pin and five-pin K-wire fixation at the three-month follow-up; second, that there is no significant difference in functional outcomes (DASH scores) between the two fixation methods at the three-month follow-up; and third, that there is no significant difference in complication rates (pin tract infection, loosening) between the two techniques.

Study population

Seventy individuals diagnosed with distal radius fractures and referred to the outpatient service consented to participate. An a priori power analysis was conducted using G*Power 3.1 software (Heinrich Heine Universität Düsseldorf, Germany), assuming a medium effect size (Cohen's d = 0.7) for the primary outcome of radial height difference between groups, with alpha set at 0.05 and desired power of 90%. This analysis indicated a need for 35 subjects per arm to detect clinically relevant differences. Patients were assigned to either the first cohort, which received fixation using a three-pin manoeuvre, or the second cohort, which received fixation through a five-pin manoeuvre, with sequential allocation occurring at the time of enrollment.

Study criteria

Eligible participants in the analysis were adult individuals aged 18 to 70 who arrived at the center no later than seven days following a closed, either extra-articular or intra-articular, distal radius fracture characterized as AO type A, B, or C. The attending surgeon judged the fracture type suitable for, and in need of, K-wire fixation as the first-line stabilization strategy. Furthermore, a critical criterion for enrollment was the patient’s capacity and readiness to furnish a signed, informed consent.

Exclusion criteria were established to create a homogeneous study population and avoid confounding variables. Patients were excluded if they had Gustilo-Anderson type II or III open fractures, pathological fractures secondary to tumors or metabolic bone disease, or severely comminuted fractures where stable fixation with K-wires alone was deemed impossible. Additionally, individuals with pre-existing conditions that could affect wrist function or healing, such as severe osteoporosis, rheumatoid arthritis, or advanced degenerative joint disease, were excluded. Other exclusion criteria included a history of previous surgery on the ipsilateral wrist, known neurological conditions affecting upper limb function, and any factors suggesting potential non-compliance with the post-operative protocol and follow-up schedule.

Protocol development

Two standardized surgical protocols were developed for this study to ensure consistency and reproducibility. While both techniques are based on established K-wire fixation principles described in the literature [7,8], we developed detailed, step-by-step protocols with specific anatomical landmarks, wire trajectory angles, and quality control checkpoints to standardize execution across all cases. These standardized protocols represent a systematic operationalization of the techniques for comparative evaluation rather than novel surgical innovations. Protocol A (three-pin technique), based on conventional cross-pinning principles, utilized three K-wires: two inserted from the radial styloid trajectory crossing toward the proximal radial shaft through the fracture site, and a third wire inserted from the dorsal-ulnar quadrant engaging the dorsal cortex to prevent dorsal displacement. This configuration provides basic three-point fixation in the coronal and sagittal planes. Protocol B (five-pin technique), an augmented approach, incorporated five K-wires: two radial styloid trajectory wires (as in Protocol A), two additional dorsal wires inserted orthogonally to buttress the dorsal cortex and counter volar tilt, and a fifth wire placed obliquely to stabilize any comminuted dorsal fragments and resist pronation forces. This configuration aims to provide enhanced multi-planar stability through additional fixation points.

Surgical methodology

All procedures were carried out under standardized regional or general anesthesia. A single senior orthopedic surgeon (SDK) with more than 15 years of experience performed all surgical procedures to ensure consistency in technique and minimize inter-surgeon variability. A closed reduction of the distal radius fracture was achieved using gentle manual distraction and sequential fluoroscopy to confirm reduction. For Group A (three-pin fixation), the three-pin construct was employed with two K-wires driven from the radial styloid toward the proximal radial shaft, each penetrating through the fracture, and a third guided from the dorsal-ulnar quadrant to contact the dorsal cortex, stabilizing the limb and halting dorsal displacement. The protocol prioritizes minimal incision and soft-tissue preservation, relying on the inherent mechanical advantage of opposing K-wires in the coronal plane. For Group B (five-pin fixation), two crossing K-wires were entered in a radial-styloid trajectory to control fracture compression, and an orthogonal pair was advanced through the dorsal aspect to brace the dorsal bone and counter acutely presented volar tilt. The fifth K-wire was inserted at an oblique angle to buttress any comminuted dorsal rim or to counteract forearm pronation forces. The augment fortified the overall construct stiffness, and the construct was assessed radiographically for correction of tilt, scapholunate angle, and fracture alignment. This technique aims to provide superior biomechanical stability. In both groups, the K-wires were left protruding through the skin, bent, and cut short. A short arm cast or splint was applied for post-operative immobilization. Post-operatively, patients were instructed on pin-site care. Follow-up was scheduled at two weeks, six weeks, and three months. K-wires were typically removed at four to six weeks, depending on radiographic evidence of healing.

Outcome measures

Patient outcomes were assessed using a three-pronged approach. Radiological outcomes were evaluated using standard anteroposterior (AP) and lateral wrist radiographs obtained at the final three-month follow-up. Three key radiological parameters were measured independently by two blinded observers (orthopedic residents not involved in patient care) to ensure reliability and minimize observer bias: radial height (the vertical distance between lines perpendicular to the long axis of the radius from the tip of the radial styloid and the ulnar corner of the lunate fossa), radial inclination (the angle between a line connecting the radial styloid tip to the ulnar corner of the distal radius and a line perpendicular to the long axis of the radius), and volar tilt (the angle between a line along the distal radial articular surface and a line perpendicular to the long axis of the radius on the lateral view) [11,12]. Interobserver reliability was assessed using the intraclass correlation coefficient (ICC), which demonstrated excellent agreement for all three parameters (ICC > 0.85 for all measurements). Loss of reduction from the immediate post-operative films was also noted.

Functional recovery was quantified using the DASH questionnaire. The DASH score is a validated, 30-item, patient-reported outcome measure that assesses physical function and symptoms in people with musculoskeletal disorders of the upper limb [13]. Scores range from 0 (no disability) to 100 (severe disability). This was administered by an independent research assistant blinded to the treatment allocation at the three-month follow-up visit.

Complications were uniformly documented at each follow-up assessment, with specific attention to pin tract infections evaluated according to the Checketts-Otterburn classification, pin loosening or migration, complex regional pain syndrome, tendon irritation or rupture, and any iatrogenic neurovascular injury [14].

Statistical analysis

The data were subjected to comprehensive statistical analysis using IBM SPSS Statistics, version 25.0 (IBM Corp., Armonk, USA). Continuous variables encompassing demographic characteristics and both radiological and functional scores were expressed as means ± standard deviations, with independent samples t-tests employed to compare the means between the two patient cohorts. Categorical variables, including the frequency and type of complications as well as fracture morphology, were summarized as frequencies and percentages; comparisons were performed using the chi-square test or, when appropriate, Fisher’s exact test. A significance threshold of p < 0.05 was applied uniformly across all analytical procedures.

Ethical consideration

The research complied fully with the ethical standards established by the Declaration of Helsinki as well as the 2017 Indian Council of Medical Research guidelines. The project protocol was subjected to and subsequently secured formal endorsement by the Institutional Ethics Committee (Human Studies) of GIMSR, Visakhapatnam. Each prospective participant was given a comprehensive briefing that outlined the study’s objectives, methodological steps, foreseeable risks, and anticipated advantages. Prior to any study-related procedures, written informed consent was procured from everyone. Confidentiality and anonymity of participants were scrupulously protected and preserved for the duration of the study.

## Results

A total of 70 patients were enrolled, completed the entire three-month follow-up, and were allocated equally to two treatment arms, comprising 35 individuals assigned to the three-pin fixation cohort (Group A) and 35 allocated to the five-pin fixation cohort (Group B). Baseline demographic data and initial fracture characteristics were balanced, with the statistical analyses confirming the absence of significant differences, thereby establishing a robust comparative paradigm (Table 1). The mean age recorded at enrollment was 45.3 years for individuals in Group A and 46.8 years for those in Group B. The distribution of the AO fracture classification was homogeneous between the cohorts; Type A fractures predominated, followed in frequency by Type C and Type B lesions, respectively.

**Table 1:** Demographic and baseline characteristics of study participants

Characteristic	Group A (three-pin, n=35)	Group B (five-pin, n=35)	p-value
Mean age (years)	45.3 ± 12.1	46.8 ± 11.5	0.58
Gender (male:female)	19:16	21:14	0.62
AO fracture type			0.81
Type A	18 (51.4%)	19 (54.3%)	
Type B	6 (17.1%)	5 (14.3%)	
Type C	11 (31.4%)	11 (31.4%)	

At the three-month follow-up, radiological assessment revealed a statistically significant advantage for the five-pin fixation technique in maintaining anatomical reduction. As detailed in Table 2, Group B demonstrated significantly better preservation of volar tilt (t = 2.10, p = 0.04) and radial height (t = 2.25, p = 0.03) compared to Group A. While radial inclination was also better maintained in the five-pin group, the difference did not reach statistical significance (t = 1.71, p = 0.09). The loss of reduction, defined as a change of >10° in dorsal tilt, >5° in radial inclination, or >2 mm of radial shortening from immediate post-operative radiographs, was observed in eight patients (22.8%) in Group A compared to only three patients (8.5%) in Group B (χ² = 3.29, p = 0.07).

**Table 2:** Radiological, functional, and clinical outcomes at the three-month follow-up DASH score: Disabilities of the Arm, Shoulder and Hand score

Outcome measure	Group A (three-pin, n=35)	Group B (five-pin, n=35)	Test statistic	p-value
Radiological outcomes				
Radial height (mm)	8.9 ± 1.8	10.5 ± 1.5	t = 2.25	0.03
Radial inclination (°)	19.5 ± 2.4	20.8 ± 2.1	t = 1.71	0.09
Volar tilt (°)	5.1 ± 2.9	8.2 ± 2.5	t = 2.10	0.04
Functional outcome				
DASH score	14.8 ± 5.6	13.5 ± 6.1	t = 0.94	0.35
Complications				
Pin tract infection	3 (8.5%)	8 (22.8%)	χ² = 3.91	0.048
Pin loosening	1 (2.8%)	2 (5.7%)	Fisher’s exact	0.55

Despite the superior radiological results in the five-pin group, this did not translate to a superior functional outcome at the three-month mark. The mean DASH score for Group A was 14.8, compared to 13.5 for Group B, a difference that was not statistically significant (t = 0.94, p = 0.35). Both groups showed a satisfactory functional recovery, with mean scores indicating only mild residual disability.

The most striking difference between the two groups was in the rate of complications, specifically pin tract infections. As shown in Table 2, the incidence of pin tract infections (Checketts-Otterburn Grade 1 or 2) was significantly higher in the five-pin group, affecting eight patients (22.8%), compared to only three patients (8.5%) in the three-pin group (χ² = 3.91, p = 0.048). All infections were superficial and resolved successfully with oral antibiotics and local wound care without requiring premature pin removal. Two patients in Group B reported significant discomfort and soft tissue irritation related to the pins, necessitating early removal at four weeks, whereas no such complaints were registered in Group A. No instances of tendon rupture, iatrogenic nerve injury, or complex regional pain syndrome were recorded in either group during the study period.

Reference values and standardization

Normal reference values for radiological parameters in the adult population are as follows: radial height 11-12 mm, radial inclination 22°-23°, and volar tilt 11°-12° [11,12]. While there are minor variations in these parameters based on sex, age, and ethnicity, the change in these parameters from post-operative to follow-up radiographs (loss of reduction) was our primary radiological outcome rather than absolute values. This approach minimizes the impact of baseline anatomical variations. Each patient served as their own control, with measurements compared to immediate post-operative radiographs. The measurement technique using standardized anatomical landmarks (ulnar corner of the lunate fossa, radial styloid tip, distal radial articular surface, and perpendicular lines to the long axis of the radius) ensures that measurements are anatomically standardized regardless of patient sex or hand dominance. All measurements were performed using digital radiography software with calibrated measurement tools.

**Figure 1:**
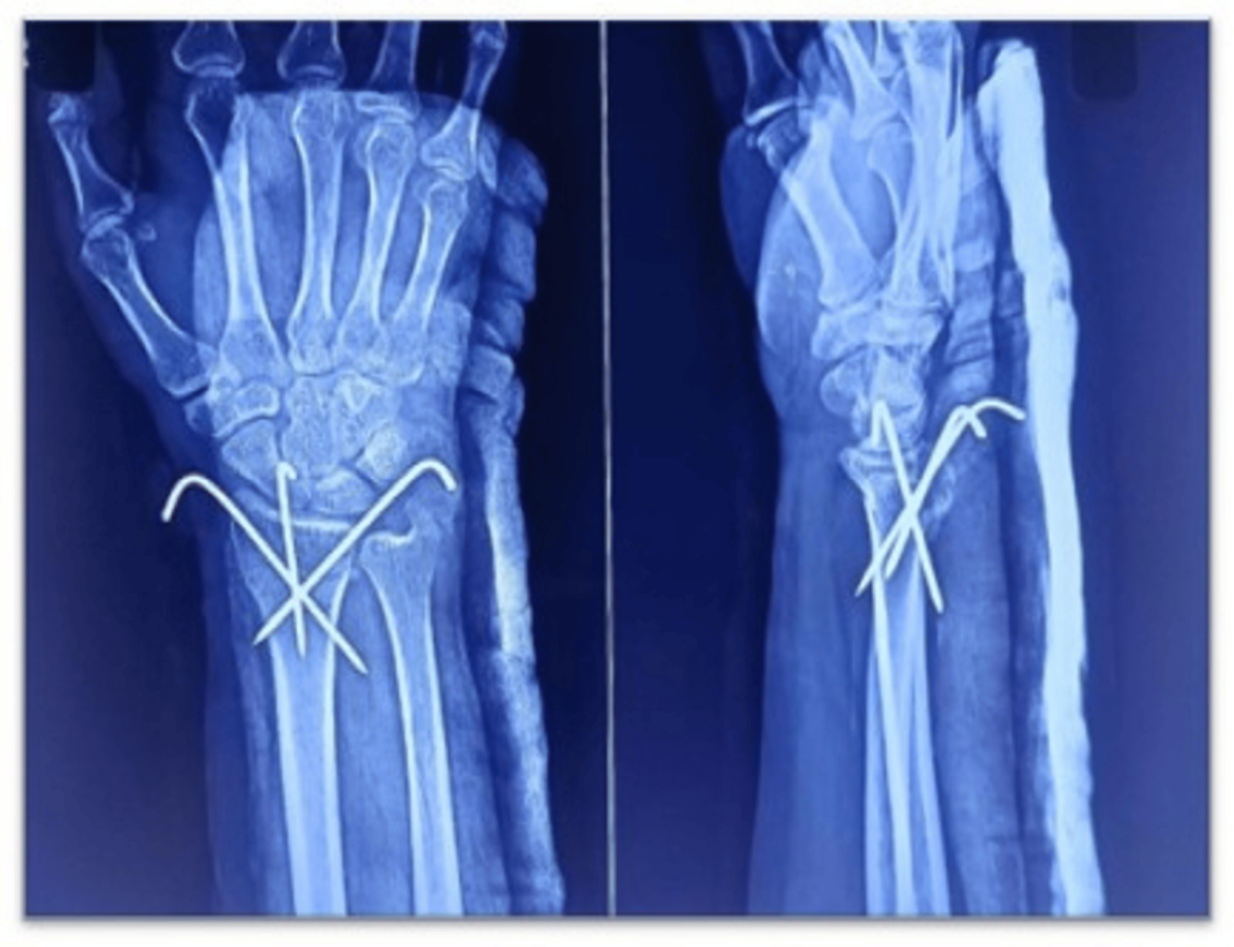
Post-operative anteroposterior and lateral radiographs of a distal radius fracture in a 36-year-old male patient treated with three-pin K-wire fixation showing adequate reduction and alignment

**Figure 2:**
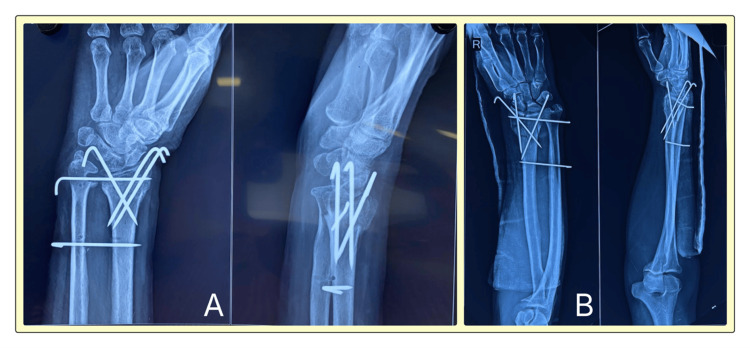
Post-operative anteroposterior and lateral radiographs of distal radius fractures treated with five-pin K-wire fixation in (a) a 54-year-old female patient and (b) a 35-year-old male patient, demonstrating enhanced stability with the multiple pin construct

The radiological images presented in Figure 1 demonstrate a typical three-pin fixation construct with adequate fracture reduction and alignment, while Figure 2 shows the more extensive five-pin configuration providing enhanced stability but at the cost of increased hardware complexity. These images illustrate the technical differences between the two approaches and their respective advantages and limitations in maintaining fracture reduction.

## Discussion

The optimal management of distal radius fractures, with the central goal of balancing stable fracture fixation with the minimization of treatment-related morbidity, continues to be a dynamic field of orthopedic research [15]. This prospective study was designed to contribute to this debate by directly comparing a simple three-pin K-wire construct with a more robust five-pin construct. Our results present a nuanced picture: the five-pin technique provides a biomechanically superior fixation, as evidenced by significantly better maintenance of key radiological parameters, but this advantage comes at the cost of a higher complication rate and does not confer a demonstrable short-term functional benefit.

The finding that the five-pin group maintained superior radiological alignment, particularly volar tilt, is consistent with biomechanical principles. The addition of extra K-wires, especially when placed to buttress the dorsal cortex, creates a more rigid, multi-planar construct that is better able to resist the deforming forces of surrounding muscles and ligaments [16,17]. This enhanced stability is particularly crucial in fractures with dorsal comminution, which are prone to collapsing back into dorsal angulation [18]. Our results, which show significantly better preservation of volar tilt and radial height (Table 2), align with previous biomechanical studies that have demonstrated the increased stiffness of multi-pin fixation constructs [19,20].

Perhaps the most clinically significant finding of this study is the discordance between radiological and functional outcomes at three months. Despite the statistically superior radiographs, the mean DASH scores for the five-pin group were not significantly better than those for the three-pin group. This finding is critical and echoes a recurring theme in the upper extremity trauma literature: the "radiograph-function paradox" [21]. It suggests that, within a certain range of acceptable alignment, the short-term patient-reported function may be more heavily influenced by factors other than precise anatomical restoration, such as soft tissue healing, pain, rehabilitation adherence, and patient biology [22]. It is plausible that the increased soft tissue irritation and discomfort associated with the five-pin technique may have negated any potential functional gains from the improved alignment in the early recovery phase. A longer term follow-up would be necessary to determine if the superior radiological alignment in the five-pin group eventually translates into better long-term function and a lower incidence of post-traumatic arthrosis.

The clear downside of the five-pin technique identified in our study was the significantly higher rate of pin tract infections. With more wires traversing the skin and soft tissues, there is an inherently greater number of potential portals for bacterial entry [23]. This, combined with potentially increased soft tissue irritation and a longer operative time, likely contributed to the 22.8% infection rate observed in Group B. While all infections in our cohort were superficial and managed successfully, they represent a significant source of patient morbidity, requiring additional clinic visits and antibiotic courses, and increased anxiety for the patient. This finding aligns with studies that have correlated the number of percutaneous implants with infectious complications [24]. The goal of any surgical intervention is to solve one problem without creating another, and a nearly one-in-four risk of infection is a substantial drawback that must be weighed heavily against the benefits of the technique.

This study has several limitations that must be acknowledged. First, the use of sequential, non-randomized allocation rather than true randomization introduces potential allocation bias and limits the strength of causal inferences, although baseline characteristics were well-matched between groups. Second, the follow-up period of three months is relatively short. While this is sufficient to assess early functional recovery and complications, it is inadequate for evaluating long-term outcomes such as the development of osteoarthritis or late fracture collapse. Third, the sample size of 70 patients, while providing sufficient power for our primary outcomes, is still modest and may not have been large enough to detect smaller differences in less common complications. Fourth, this was a single-center study with all surgeries performed by a single experienced surgeon, and the results may be influenced by local surgical preferences and rehabilitation protocols, potentially limiting their generalizability. Finally, while outcome assessors for radiographic measurements and DASH scores were blinded to treatment allocation to minimize observer bias, the patients themselves could not be blinded to the number of pins used, which may have influenced patient-reported outcomes.

## Conclusions

In our cohort of 70 patients with distal radius fractures, five-pin K-wire fixation provided statistically superior maintenance of volar tilt (8.2° vs. 5.1°, p = 0.04) and radial height (10.5 mm vs. 8.9 mm, p = 0.03) compared to three-pin fixation at the three-month follow-up. However, this radiological advantage did not translate to superior functional outcomes, with similar DASH scores between groups (13.5 vs. 14.8, p = 0.35). Importantly, five-pin fixation was associated with a significantly higher pin tract infection rate (22.8% vs. 8.5%, p = 0.048). Based on these findings from our study population, the routine application of five-pin fixation for all distal radius fractures cannot be recommended. Our data suggest that three-pin fixation provides adequate stability with lower complication risk for most fracture patterns. The five-pin technique may offer benefit in specific cases where enhanced mechanical stability is critical, though this should be weighed against increased infection risk. Randomized controlled trials with longer follow-ups are necessary to determine if early radiological advantages result in long-term functional benefits or reduced arthritis rates.
